# Nursing students’ experiences of clinical competency evaluation in a pre-registration nurse education programme: A qualitative study

**DOI:** 10.4102/curationis.v48i1.2699

**Published:** 2025-04-15

**Authors:** Joseph Sitwira, Daniel O. Ashipala, Vaja Katjimune

**Affiliations:** 1 Department of General Nursing Sciences, School of Nursing and Public Health, Faculty of Health Sciences and Veterinary Medicine, University of Namibia, Rundu, Namibia

**Keywords:** evaluation, experience, nursing students, pre-registration, programme, clinical competence

## Abstract

**Background:**

The evaluation of clinical competence is a diverse, rigorous procedure that determines a student’s clinical competence. Despite this, little research exists on nursing students’ experiences with clinical competency evaluations in Namibia. Nurse educators should thus explore nursing students’ experiences of clinical competency evaluations in order to establish what challenges they face, as this can be beneficial for nurturing a positive learning environment.

**Objectives:**

This research assesses nursing students’ experiences of clinical competency evaluations in a pre-registration nurse education programme at the Faculty of Health Sciences in Namibia, University of Namibia, Rundu campus.

**Method:**

The study was conducted at a public nurse education institution in Namibia. A qualitative approach was employed utilising an exploratory, contextual and descriptive design. This study was conducted from August 2023 to October 2023 among (second-, third- and fourth-year) nursing students who were enrolled for a Bachelor of Nursing Science (Clinical) (Honours) degree. Data were collected through semi-structured interviews with 16 nursing students being selected using a convenience sampling method. All interviews were audio recorded and transcribed verbatim. A thematic analysis method was utilised to analyse the data.

**Results:**

Three themes emerged in this study: (1) positive experiences of evaluation for clinical competence; (2) negative experiences of evaluation for clinical competence and; (3) recommendations to improve the clinical evaluation of competence.

**Conclusion:**

The results showed that nursing students had both negative and positive experiences during the clinical assessment process. The positive experiences included integrating theory and practice, while negative experiences included the poor attitudes of the clinical evaluators and limited time.

**Contribution:**

The results of this study can be used to develop targeted interventions and strategies to improve the challenges students encounter during clinical assessment.

## Introduction

Nursing education incorporates both theoretical education and clinical practice. Practical lessons are taught in a clinical learning environment, for example, clinics, health centres and hospitals, which increases students’ knowledge and understanding of a real nursing environment. The combination of academic and practice learning is a feature of pre-registration nursing programmes globally (Price et al. 2011). Lecturers and preceptors (known in some nations as practice facilitators, instructors, practice assessors or supervisors) play an important role in the development of professional skills and shaping nursing (Soroush et al. 2021), and they are responsible for equipping undergraduate nursing students with competent instruction in the classroom and effective assessments during clinical practice. It is crucial that nursing students are adequately prepared to provide high-quality patient care and service (Al Mutair & Redwan 2016; Massey, Chaboyer & Anderson 2017). It is thus essential that nursing students be assessed for their clinical competency in order to determine the success of their clinical training, the learning process and the level of teaching (Alkhelaiwi et al. 2024), as this determines their readiness to offer safe and effective patient care (Jeffries 2012).

Clinical competence can be described as those actions required to fulfil nursing care (Fukada 2018). Clinical evaluation seeks to assess students’ problem-solving, understanding, knowledge, attitudes, ethics and technical skills (Msiska et al. 2015). According to these authors, clinical evaluation helps to maintain standards and protects the public by making sure that graduates from nursing programmes have learned the required skills and are able to practice safely. In addition, Zhu et al. (2017) stated that clinical competency evaluation entails assessing nursing students’ ability to apply theory to real-world clinical circumstances through assessments such as skills demonstrations, objective structured clinical examinations objective structured clinical examinations (OSCEs) and clinical simulations. These assessments are used to gauge a nursing student’s clinical judgment, communication skills critical thinking abilities, and ability to offer comprehensive patient-centred care (Turner 2017).

Research indicates that in clinical evaluations, students must exhibit ‘appropriate professional behavior, establish an appropriate interaction with patients, prioritize problems, have basic knowledge about clinical methods, perform the care procedures correctly, and apply critical thinking’ (Hala et al. 2016:9). A study by Wu and Al (2018) also highlighted the importance of nursing students understanding their experiences with clinical competency evaluations, as this process not only affects their educational journey but also has far-reaching ramifications for the quality of patient care. Clinical assessment thus aims to prepare nursing students to become safe, responsible and ethical nurse practitioners. This involves clinical judgement, technical proficiency, communication, critical thinking aend ethical decision-making (Jeffries 2012). Students’ competence is assessed by sending students to clinical practice with logbooks that record demonstrators’ and evaluators’ signatures. Demonstrators and evaluators play a critical role in executing the OSCE, which includes contributing to the design of OSCE stations, identifying competencies to be tested and providing individual or group feedback to students after examination (Majumder et al. 2019). There are also selected clinical procedures that must be taught and evaluated by lecturers for students’ class marks.

In Namibia, students are expected to score an average of not less than 50% to pass an objective structured clinical examinations (OSCE), yet Hala et al. (2016) stated that nursing students face a variety of challenges in clinical practice, including a lack of time, inadequate facilities and a lack of knowledge of scientific functions (Niaz et al. 2019). Over the years, nursing education has transitioned from an apprenticeship-based approach to a formal, structured curriculum that combines didactic instruction and clinical experiences. The evaluation of clinical competence in particular is a diverse and rigorous procedure in modern nursing education (Benner 2016). Nowadays, nursing students expect to receive thorough practical training to acquire the necessary skills and knowledge needed to fulfil the demands of current healthcare settings and to become clinically competent. The subjective nature of clinical competence assessments sometimes presents difficulties, especially when the criteria for evaluation are not clearly specified, and assessors may bring their own prejudices and preconceptions to the process. This subjectivity results in inconsistent evaluations, which then leads to disparities and unfairness in grading (Zanga & De Gioannis, 2023).

Nursing students’ experiences during an evaluation process can be complex and influenced by a variety of factors, which can cause anxiety and tension. The high-stakes nature of these examinations, along with the duty of caring for actual patients, creates a difficult learning environment (Yonge et al. 2020). Evaluations can also impact nursing students’ self-confidence and self-esteem (Jeffries et al. 2012), while demographic characteristics, prior clinical exposure and individual learning styles may all influence students’ experiences with clinical competency evaluations (Kuipers et al. 2019). At the University of Namibia (UNAM), nursing students are assessed equally on theory and practice throughout their studies (Shatimwene, Ashipala & Kamenye 2020). At the practical level, student nurses must undergo assessments by faculty and clinical instructors to evaluate their level of competence. Investigating nursing students’ experiences of clinical evaluation can thus shed light on how different backgrounds and learning styles influence the evaluation process.

## Research methods and design

The researcher utilised a qualitative research design that was descriptive, contextual and explorative to investigate the experiences of nursing students regarding clinical competency evaluations (Polit & Beck 2019). The semi-structured, individual interviews with 16 nursing students enabled the researcher to comprehend the lived experiences of the interviewees. The study seeks to answer the following two research questions as namely: (1) What is the experience of nursing students with clinical competency evaluation? and (2) What recommendations should be made to improve the clinical competency evaluation?

All the nursing students who took part in the study broadly display the demographics as the wider population. The researcher who conducted the interviews was a fourth-year nursing student and had an existing relationship with all the interviewees. This relationship did not have any influence on the research findings as there was no power imbalance between the researcher and interviewees.

### Participants and setting

This research was undertaken at a public university in the Kavango East region, Namibia. The accessible population included undergraduate nursing students (second, third and fourth year) who were enrolled for a Bachelor of Nursing Science (Clinical) (Honours) degree at the School of Nursing and Public Health in 2023. A total of 16 participants were chosen via the convenience sampling method. The participant inclusion criteria included: (1) full-time second-, third- and fourth-year undergraduate nursing students enrolled for the degree of Bachelor of Nursing Science (Clinical) (Honours); (2) willingness to participate and (3) availability.

### Data collection

The researcher collected data in August 2023 to October 2023 during individual interviews under his research supervisors’ guidance. The interviewees signed their agreement to take part in the study with the understanding that they could withdraw at any time with no negative consequences. Before actual data collection was conducted, pilot interviews were undertaken with three interviewees from the same sampling unit to assess the appropriateness and effectiveness of the interview guide, as well as to determine if the interviewees encountered any challenges. The pilot interviews comprised of two males and one female participant. A few adjustments were made to the interview guide following this. Findings from the pilot interviews were not incorporated into the final study. The interview guide was developed based on the research objectives, research questions and the literature review. Although the researcher is a novice researcher, he was trained in qualitative research interviewing techniques and conducted a pilot interview with nursing students from the same sampling unit to ensure that the interview process was of high quality and yielded meaningful data. During the actual qualitative data collection, the supervisor for the researcher was available to assist the researcher as needed. The semi-structured interviews, which lasted between 40 and 50 min, were held at a venue that was convenient for the interviewees. During the interviews, which were conducted according to an interview guide, the researcher recorded each interview session the interviews. Saturation was reached after 16 interviews. The authors partly developed the interview guide based on the research questions, generating two key questions. The additional questions were probing questions that were based on the responses of the participants:

1. Tell me more about your experiences as a nursing student with regard to clinical competency evaluation?
2. Can you tell me what recommendations should be made to improve the clinical competency evaluation?

### Data analysis

The researcher used an inductive approach using the thematic analysis technique to analyse the data, which were transcribed verbatim from the audio recordings. The data were analysed according to Braun and Clarke’s (2019:295). Systematic six-step process, which included: ‘(1) becoming familiar with the data; (2) generating initial codes; (3) coding data; (4) determining and reporting themes; (5) defining and naming themes; and (6) interpreting the data’.

### Data trustworthiness

The trustworthiness of the research was ensured by adhering to the criteria put forward by Lincoln and Guba (1965), that is, dependability, transferability, credibility and confirmability. Both the dependability and the confirmability of the research were ensured through the use of an experienced independent coder. Each analyst conducted their own thematic analysis, and any discrepancies or potential biases were discussed in detail. Decisions were reached through consensus to ensure a thorough and unbiased interpretation of the data. This multi-layered approach helped to enhance the validity and credibility of the data analysis process. After initial coding, the researcher met with the co-coder (the data analyst) to discuss the findings from the perspectives of the co-coder and the researcher. There was general agreement on key issues surrounding the codes, sub-themes and themes. The final wording of the findings did not exactly match the initial findings or the findings from the co-coder. However, the key aspects were retained, and these findings matched most of the sub-themes from the co-coder. Any differences can be attributed to the researcher’s condensing of the themes and further aligning them to the data. An in-depth recording of all evidence was also kept. The transferability of the research was ensured through a confirmability audit, which was conducted independently by an expert researcher. Lengthy and varied engagement with the interviewees ensured the study’s confirmability, as the researcher engaged in reflexive practice throughout the data analysis process, regularly reflecting on his own biases, assumptions and preconceptions that had the potential to influence data interpretation.

### Ethical considerations

Approval to conduct the study was received from the University Namibia’s Research Ethics Committee and the Ministry of Health and Social Services (Reference no: 23/4/2/3), as was ethical clearance to collect data (School of Nursing Ethics Committee [SoNPHEC]: reference no.: 95/2023). Written informed consent was obtained from all participants before participating in the study, including audio recordings of interviews. The informed consent also informed the participants that they could opt not to sign it or withdraw at any time without penalties. Furthermore, they were able to decide on the date, place and time of their interview. The possibility existed that some participants might experience discomfort or become emotional during the interviews. Therefore, provision was made for participants to consult a social worker at the Department of Social Welfare should this have been necessary. This sensitivity to the participants’ feelings was aligned with the right to self-determination. The interviewees were assigned codes to ensure their anonymity and confidentiality, and all data were kept on a password-protected computer. These data will be disposed of according to the university’s policy.

## Results

### Socio-demographic description of study participants

The study participants were undergraduate nursing students in the Bachelor of Nursing Science (Clinical) (Honors) programme at the public university in Kavango East, Namibia. Each interviewee was under 30 and was a second, third or fourth-year student. All the nursing students who took part in the study broadly display the demographics as the wider population. The demographic data of the participants are presented in Table 1.

**TABLE 1:** Characteristics of study participants.

Characteristics	Frequency
**Age (years)**	
18–22	9
23–30	7
**Gender**	
Male	6
Female	10
**Marital status**	
Single	11
Married	5
**Year of study**	
Second	4
Third	5
Fourth	7

The three themes that emerged from the data analysis (as indicated in Table 2) are as follows: (1) positive experiences of evaluation for clinical competence, (2) negative experiences of evaluation for clinical competence and (3) recommendations to improve the clinical evaluation of competence.

**TABLE 2:** Themes and sub-themes.

Themes	Sub-themes
1. Positive experiences of evaluation for clinical competence	1.1 Theory-practice integration 1.2. Build good communication skills 1.3. Nervousness and tension reduced
2. Negative experiences of evaluation for clinical competence	2.1 Attitudes of clinical evaluators 2.2. Limited equipment 2.3. Limited time for the procedures 2.4. Unconducive environment for clinic evaluation 2.5. Two-week block system 2.6. Clinical evaluation feedback
3. Recommendation to improve the clinical competency evaluation	3.1 Peer group teaching 3.2. Regular clinical accompaniment of students 3.3. Extend the current 2-week to a 4-week block system 3.4. Provision of constructive feedback 3.5. Recruit more clinical instructors 3.6. Assistance of the university to provide clinical equipment

### Theme 1: Positive experiences of evaluation for clinical competence

This theme provided a description of the participants’ positive experiences regarding the evaluation of clinical competence. Positive experiences refer to evaluation experiences that had a successful outcome, those that may not have had a successful outcome, but that were considered valuable learning experiences by the student and experiences in which the evaluation process went smoothly. Three sub-themes emerged from this theme: (1.1) theory-practice integration, (1.2) building communication skills and (3.3) nervousness and tension reduction.

#### Sub-theme 1.1: Theory-practice integration

This sub-theme focussed on the participants’ positive experiences of how clinical evaluation helped them effectively apply their theoretical knowledge in real-world situations:

‘Clinical evaluation offers valuable learning opportunities, allowing me as a student to apply my theoretical knowledge to a real patient care situation.’ (P3, female, fourth year)

‘I might say that every assessment that we usually do, they provide us with a demo, and they give you enough time for you to practice before the real assessment is conducted before you get marked. And this contributes to the passing rate where we tend to get more marks on the practical part compared to theoretical. And I would generally believe that practical is something that you need to perform better than theory.’ (P2, female, second year)

#### Sub-theme 1.2: Build good communication skills

This sub-theme emphasised the participants’ development of effective communication skills, highlighting that the evaluation helped them enhance their communication skills:

‘I have developed new communication skills with the nurses and the patients.’ (P11, male, fourth year)

‘And also, it taught me how to communicate with my fellow colleagues and also in the community where I am, or sometimes to my patients because communication helps creating a bond between you as a nurse and your patients.’ (P4, male, fourth year)

#### Sub-theme 1.3: Nervousness and tension reduced

In this sub-theme, the data revealed that throughout the years of doing clinical competency evaluations, the participants had gained confidence:

‘It boosted my self-esteem, to be confident enough in what I’m doing, to believe in myself: yes, I can do it. So, this really helped me to boost my self-confidence and self-esteem.’ (P4, male, fourth year)

‘Now I’m used to interacting with the lecturers and doing the assessments, so I don’t really feel nervous.’ (P8, female, third year)

### Theme 2: Negative experiences of evaluation for clinical competence

The participants described their challenges or negative experiences during clinical competence evaluations. Negative experiences refer to evaluation experiences for those that have not had a successful outcome, but that were considered invaluable learning experiences by the student. The sub-themes that emerged from this theme were: (2.1) attitudes of clinical evaluators, (2.2) limited equipment, (2.3) limited time for the procedures, (2.4) Unconducive environment for clinical evaluation, (2.5) a 2-week block system and (2.6) clinical evaluation feedback.

#### Sub-theme 2.1: Attitudes of clinical evaluators

In this sub-theme, the participants described challenges regarding the negative attitudes of clinical evaluators and nurses during evaluations:

‘In some of the evaluators that I have observed, the moment you introduce yourself, the evaluator is already writing final notes without you doing the whole procedure and that really makes me lose hope.’ (P2, female, second year)

‘Those evaluators, they always have bad attitudes. You walk in the room, they don’t greet you. They make you feel nervous, then later on, you start forgetting what you even studied because even just the way they look at you, you feel intimidated.’ (P12, male, fourth year)

#### Sub-theme 2.2: Limited equipment

This sub-theme described how the limited equipment or resources affect the participants’ clinical competency:

‘Lack of equipment is also a major problem. I can remember this year when we were doing CPR, some materials were not there. It happened that the groups which are rehearsing that procedure, CPR, they are 17 groups, so it was delaying time. We were having 20 minutes per group.’ (P15, female, second year)

‘I haven’t used the material before. So, I’ve only heard it or seen it theoretically, but handling it with my physical hands, I think most of our work is practical. So, we need to do those things with hands.’ (P9, male, second year)

#### Sub-theme 2.3: Limited time for the procedures

This sub-theme focussed on the participants’ time challenges. Many respondents stated that assessment time is limited and there are few instructors assessing the students:

‘Not enough time, so it affected me very badly because I did not cover some of the important part, so it affected my competency with which I could learn something new.’ (P13, male, third year)

‘With a lack of time, it affected me in a bad way with some of the procedures because I didn’t even finish the procedure or it made me speed up my procedure. So, it even resulted in me getting low marks because I sped up the procedure.’ (P11, male, fourth year)

#### Sub-theme 2.4: Unconducive environment for clinic evaluation

Many interviewees commented on the negative environment, which made it challenging to give demonstrations or perform the procedures:

‘The learning environment is not conducive. Let me talk about our simulation part. It’s not really conducive.’ (P4, male, fourth year)

‘The overcrowding makes it hard for me to learn or to see what my lecturer is trying to show to me. So sometimes I might be left out because maybe I’m at the back and I can’t see what is being done to the doll.’ (P10, male, second year)

#### Sub-theme 2.5: Two-week block system

In this sub-theme, the respondents indicated that the 2-week block for the clinical setting is not sufficient and that affects their competency as they get less time in a particular ward, which sometimes leads to incomplete logbooks:

‘The two weeks, it’s not enough because you’ll be there in the ward and maybe it’s your first time. That first week is just for orientation and then the second week is when you start doing things after demonstrations.’ (P5, female, third year)

‘The duration of allocation at a certain department or ward is not enough regarding especially, like, let me say you’re allocated in theatre. In theatre, we’re only allocated during our third year. Okay. So, it’s really bad because it really affects our experience or competency to say. Imagine it’s your first time to be allocated there, and you’re only allocated once in a year for two weeks.’ (P4, male, fourth year)

#### Sub-theme 2.6: Clinical evaluation feedback

This sub-theme focussed on the evaluation feedback to nurses and preceptors:

‘Lecturers they expect us to come back with feedback … but sometimes the nurses are just stingy with their knowledge.’ (P9, male, second year)

‘They only come to assess, to evaluate us when it comes to our continuous assessment for us to get the CA marks, which is not so good because we need to be more competent and we need to continue being evaluated throughout the year. But you find that the only time they will only come to evaluate is during the time that they will need you to have that mark for you to qualify for exams.’ (P1, female, fourth year)

### Theme 3: Recommendation to improve the clinical competency evaluation

This theme described what the interviewees said when they were asked for recommendations for improving the clinical competency evaluation. Seven sub-themes emerged: (3.1) peer group teaching, (3.2) regular clinical accompaniment of students, (3.3) extension of the 2-week block system to 4 weeks, (3.4) constructive feedback, (3.5) more clinical instructors recruited, (3.6) assistance of the university to provide clinical equipment and (3.7) proposal of clinical simulation at the campus.

#### Sub-theme 3.1: Peer group teaching

In this sub-theme, the interviewees suggested that faculty should promote mentorship and provide an online forum:

‘I should always have mentorship as a student. I should always promote mentorship programs whereby experienced nursing students can guide and support nursing students in their clinical learning.’ (P10, male, second year)

‘… online forum, must have it with our lecturers and our head of the department so we rise of a complaint.’ (P5, female, third year)

#### Sub-theme 3.2: Regular clinical accompaniment of students

This sub-theme described the participants’ opinions regarding students being accompanied at clinical practice by the preceptors or a clinical instructor to monitor their progress:

‘They must make sure that nursing students are accompanied in the clinical area on a daily basis; if they are there then we can ask for a demonstration from them or we can give feedback to them.’ (P6, female, fourth year)

‘They should at least be with us throughout the year and help us to do all these procedures and be evaluated on them, for them to know really this student is competent, because at the end of the day, you know, I’m a producing student.’ (P1, female, fourth year)

#### Sub-theme 3.3: Extend the current 2-week to a 4-week block system

This sub-theme addressed recommendations that the School of Nursing extend its block system to 3 or 4 weeks. The participants mentioned that they need to spend more time practising and to complete their logbooks:

‘The clinical department must ensure that students are placed for a period which is sufficient for learning in the clinical settings; at least three weeks will be better than two weeks because the two weeks, it’s not enough.’ (P16, female, fourth year)

‘I think that since we are allocated in a ward for two weeks, so I’m suggesting that maybe they should increase it to three weeks, I think that would be much better.’ (P4, male, fourth year)

#### Sub-theme 3.4: Provision of constructive feedback

This sub-theme focussed on the participants’ recommendations that evaluators communicate with their students after a procedure, including how to improve their competence:

‘After we [*are*] done at the end of each procedure, a supervisor must encourage a student and give constructive feedback to the student on what has gone wrong or what the student did.’ (P1, female, fourth year)

#### Sub-theme 3.5: Recruit more clinical instructors

This sub-theme described the respondents’ recommendations regarding time management. All the interviewees argued that the university should recruit more instructors to accommodate the students during clinical evaluation. This also would afford students more time under instruction:

‘I think it’s really necessary to increase the number of the clinical inspectors, even three or four, so that the students will have enough time to do the procedure.’ (P7, female, third year)

‘They need to recruit more clinical instructors so that they can be able to accommodate a lot of students at the same time.’ (P8, female, third year)

#### Sub-theme 3.6: Assistance of the university to provide clinical equipment

This sub-theme focussed on the participants’ suggestion that the university provide clinical equipment for students to use during evaluation:

‘I suggest that the University provides us with enough equipment, then we can be able to at least practice those procedures and be evaluated on them using that equipment.’ (P14, female, third year)

‘If the university could buy materials for students, which will be strictly for students, just to be used in simulation classes, I think it will be best. And they have to ensure in a way that it covers every material that’s needed.’ (P2, female, second year)

## Discussion

This study explored undergraduate nursing students’ experiences of clinical competency evaluation at a national university in Namibia. The participants indicated that clinical evaluation offers them valuable learning opportunities that allow them to put their theoretical knowledge into practice. Similarly, Fero et al. (2015) reported that clinical assessment increases students’ ability to practice in the real world and helps nursing students make deeper connections between theoretical knowledge and patient care (Günay & Kilinç 2018). Rystedt and Gustafsson (2013) similarly noted that during clinical internships, nursing students should be exposed to different learning situations to integrate practical professional skills with theoretical knowledge. Clinical instructors play an important role in assisting students to integrate this knowledge (Voges & Frantz 2019).

The respondents also made it clear that clinical competency evaluation enhances their communication skills, which aligns with Immonen et al.’s (2019) findings that interactions between mentors, students, nurses and faculty in a clinical learning environment enhance professional development and student learning. Good communication is essential to establish trust between nurses, patients and their family members, which is the foundation of good healthcare (Anderson et al. 2019). Therefore, clinical assessment should include ways to enhance nurses’ independent thinking and training in communication while also providing alternative communication methods (Perry et al. 2021).

Some participants reported gaining confidence and reducing their nervousness during their evaluations, especially when assessed by a familiar evaluator. Kiernan (2018) reported that a person’s self-confidence is a marker of proficient performance, while a study by Perry, Henderson and Grealish (2018) found that in the final stage, students become more independent and comfortable performing nursing tasks and are eager to grow. Varutharaju and Ratnavadivel (2014) added that someone’s confidence can be improved through simulation training, as this encourages critical thinking and improves knowledge retention. He further stated that nursing students learn and gain confidence and proficiency if consistently exposed to demonstrations in which they utilise psychomotor skills and critical reasoning (Debora, Joy & Ronald 2015).

In this study, the participants complained that some instructors and nurses behaved negatively during the assessment process, which caused fear. These complaints correspond to research by Anim-Boamah, Christmals and Armstrong (2021), who stated that students are sometimes mistreated by examiners when they make mistakes during exams. In addition, Rayan (2019) noted that although some students receive professional treatment, others face negativity from examiners during an assessment, which might affect the outcome. Fisseha & Desalegn (2021) also reported that over half of students perceive examiners to be rude, hostile and uncooperative, causing them to panic during the assessment process.

The study participants indicated that there is a constant challenge with insufficient training equipment. This aligns with John (2020), who found that the OSCE stations were poorly arranged and the equipment was faulty. Similarly, Younas et al. (2019) found that although books and manuals are available to acquire skills, physical equipment and financial resources are limited.

Many respondents stated that the assessment time is limited, and there are too few instructors who may push students to complete quickly. These findings align with those of Beiranvand, Hosseinabadi and Fatemeh (2017), where students complained about the lack of time to complete the necessary procedures at each station. In a study conducted by John (2020), many students were similarly concerned about the time needed to demonstrate skills. In addition, Bradley and Postlethwaite (2003) and Mahmoud and Mostafa (2011) found that students expressed concern about limited time during assessment. A lack of time can increase anxiety and stress as students rush and confuse procedural steps (Rafati, Pilevarzade & Kiani 2020).

The block placement model was considered by some participants to offer a realistic and authentic experience of the registered nurse role in Australia, Canada and the United Kingdom, which was considered most conducive to learning (Birks et al. 2017). Very surprisingly, participants in the current study highlighted the negative impact of the limited time that 2 weeks yielded for their clinical competency evaluation. Consequently, participants in this study recommended an extended period for clinical practice, stating that the 2-week block system does not give them enough time to integrate theory into clinical practice. This is believed to have a negative effect on their clinical competency evaluation outcome (Muhora & Ashipala 2022; Shatimwene et al. 2020). Contrary to the above, Muhora and Ashipala (2022) reported in their study that a study period of 1 to 2 weeks is insufficient time for nursing students to become familiar with the clinical environment. The interviewees in this research listed concerns about their inadequate knowledge of some uncommon procedures performed in clinical settings and said that attending the theatre department once a year is not enough.

This study’s results suggest that peer group teaching among nursing students would be useful, which is as per Almalkawi, Jester and Terry (2018), who stated that mentors play an important role in providing feedback. This aligns with Tuomikoski et al. (2020), who argued that clinical supervisors should spend time supporting students in the clinical environment and guiding students through learning, training, development and assessment.

The participants also complained about not receiving proper feedback from clinical preceptors, evaluators and nurses, which corresponds with the findings of Duers and Brown (2019) who noted that feedback is given orally. By bypassing written notes, students do not receive accurate, specific, constructive and timely information. Feedback in clinical nursing skills assessment has been widely researched as it helps develop practice, identify areas requiring further development and document performance patterns, leading to improvements. It also improves skills, supports assessment systems and creates learning situations (Burke et al. 2016). Additionally, it is imperative that students clearly understand what is required to achieve the desired level of proficiency (Wu et al. 2015).

According to a study conducted by Younas et al. (2019) and Jay (2007) in parkistan, it is reported that it is crucial for the Pakistan Nursing Council, as an accreditation body to provide adequate resources to facilitate learning and teaching. Hasona (2006) similarly noted that the quality of the clinical learning environment is essential to determining the quality of experience and outcomes of qualified nursing and professional nursing students. Furthermore, John (2020) reported that an exam room must meet basic criteria regarding room temperature, ventilation, unpleasant odours and water availability.

### Strengths, limitations and areas for further research

One of the strengths of this study is that the students’ own perspectives and lived experiences were considered, which enabled deep insight into, and a broad understanding of the experiences of university students’ clinical competency evaluations. The explorative design permitted the interviewees to narrate, describe and interpret their lived experiences while offering ideas for improvement. A limitation is that the study was conducted on one campus; therefore the findings cannot be generalised across other universities or faculties. Given the researcher’s potential position as a senior nursing student at an educational institution and familiarity with the participants, there could have been a perceived power dynamic that influenced their involvement, comfort level and honesty in providing feedback. While the researcher aimed to create a neutral and open environment, it is possible that these factors could have affected their ability to be fully candid. To address this, the researcher made efforts to encourage open dialogue and ensure anonymity in feedback. The researcher acknowledges that the potential for a conflict of interest existed, which could have impacted the participants’ involvement. Further research is recommended focussing on the assessment method that can adequately measure all aspects of evaluation for clinical competence, which may include aspects such as consistency, validity, reliability and complexity.

## Conclusion

The aim of this research was to explore nursing students’ experiences during clinical competency evaluations. The study shows that student nurses face challenges during clinical evaluations, which include negative attitudes of clinical evaluators, insufficient equipment, limited time for the procedures, an unconducive environment for clinical evaluation, the 2-week block system and poor clinical evaluation feedback. This study’s results could be useful in the creation of targeted strategies and interventions to mitigate the issues faced by students during clinical evaluation.
